# Clamping down on mismatches

**DOI:** 10.7554/eLife.18365

**Published:** 2016-07-12

**Authors:** Guo-Min Li

**Affiliations:** Department of Biochemistry and Molecular Biology, Norris Comprehensive Cancer Center, University of Southern California Keck School of Medicine, Los Angeles, United Statesguominli@usc.edu

**Keywords:** mismatch repair, proliferating cell nuclear antigen, DNA replication, Xenopus egg extract, MutSα, *Xenopus*

## Abstract

A sliding clamp complex may help correct DNA replication errors by keeping track of which DNA strand is new and which is the template.

**Related research articles** Kawasoe Y, Tsurimoto T, Nakagawa T, Masukata H, Takahashi TS. 2016. MutSα maintains the mismatch repair capability by inhibiting PCNA unloading. *eLife* **5**:e15155. doi: 10.7554/eLife.15155

**Image** The PCNA sliding clamp binds to DNA asymmetrically

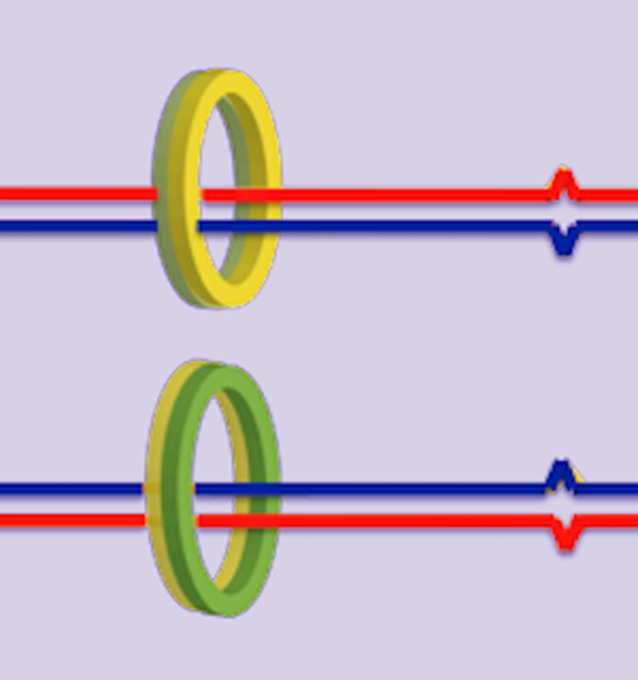

Errors made when DNA is replicated can lead to cancer and genetic disorders, so cells rely on a system called mismatch repair (MMR) to remove many of the errors from newly synthesized DNA (Hombauer et al., 2011; Kunkel and Erie, 2015; Simmons et al., 2008). These errors include mismatches that occur when the wrong DNA base is added into the new DNA strand (i.e. a base that does not match the one in the template strand). MMR must deal with these mistakes before the DNA is repackaged into nucleosomes, which would block the repair mechanisms. As such, a long-standing question in this field is how the MMR system discriminates the new strand (which contains the errors) from the template (which is error-free) both quickly and accurately.

Now, in eLife, Tatsuro Takahashi and colleagues at Osaka University and Kyushu University report a new twist in this story (Kawasoe et al., 2016). Newly synthesized strands of DNA have small gaps or nicks (Kunkel and Erie, 2015), and such signals can be used to direct MMR to a specific DNA strand in cell-free experiments (Holmes et al., 1990). However, Takahashi and colleagues – who include Yoshitaka Kawasoe as first author – found, via experiments with *Xenopus* cell extracts, that MMR can still correctly identify the new DNA strand even after any gaps or nicks had been filled in. This unexpectedly implied that there must be a second signal that allows MMR in eukaryotes to distinguish the new DNA strand from the template.

So, what is this second signal? PCNA is a ring-shaped complex that acts as a “sliding clamp” to coordinate DNA replication by traveling along the DNA template. Eukaryotes use PCNA and a mismatch recognition complex called MutSα to start MMR (Kadyrov et al., 2006; Kunkel and Erie, 2015; Umar et al., 1996), and these two complexes interact directly at the site of a mismatch (Clark et al., 1999; Flores-Rozas et al., 2000; Kleczkowska et al., 2001). Kawasoe et al. now reveal that the PCNA complex provides the secondary signal that allows the cell to “remember” which strand of DNA is which (Figure 1A,B).

**Figure 1.**
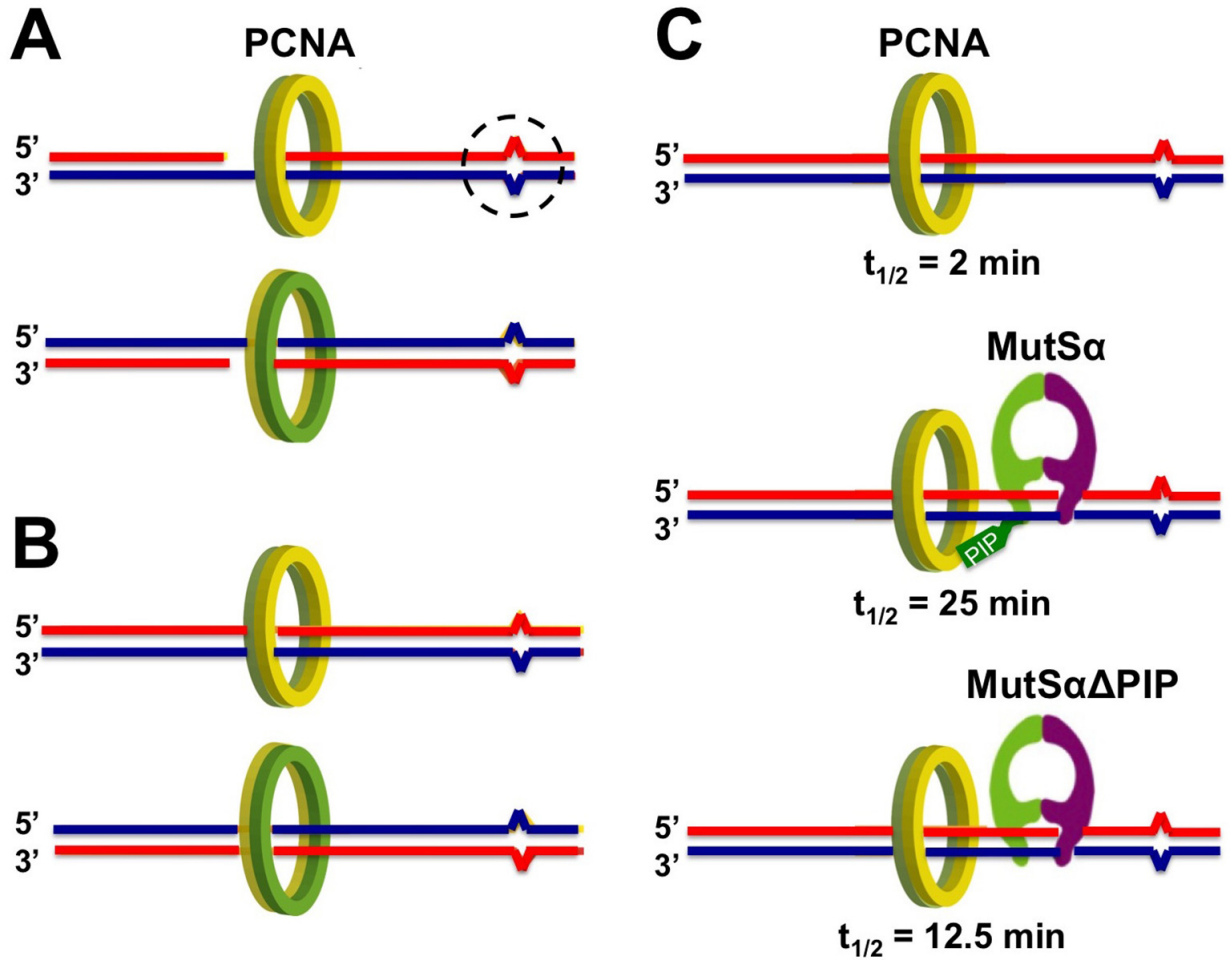
PCNA binds to DNA in an asymmetric way. Base-base mismatches (dashed circle) result when a base is added into a newly synthesized DNA strand (red) that does not match the corresponding base in the template strand (blue). (**A**) Newly synthesized strands of DNA often have small gaps that are filled in and ligated later, and the ring-shaped complex PCNA forms non-identical complexes with 5’-gapped (top) or 3’-gapped (bottom) heteroduplex DNA. Note that one side of the complex (green) always faces towards the 5’ end of the new DNA strand, while the other (yellow) faces towards the 3’-end. (**B**) The asymmetry of the PCNA-DNA complexes is conserved after the DNA gaps are filled in and ligated; this could allow DNA mismatch repair to distinguish the error-containing new strand from the error-free template. (**C**) Kawasoe et al. show that PCNA spends little time on DNA containing a mismatch in the absence of the mismatch recognition complex MutSα (top). PCNA spends much longer on the DNA when MutSα is present (middle), but not quite so long if MutSα’s PCNA interacting protein (PIP) motif is removed (MutSα∆PIP; bottom). Times given in figure are the half-lives of the PCNA-DNA complexes (t_1/2_).

The PCNA clamp loads onto DNA ends in an asymmetric way (Pluciennik et al., 2010). This means that one side of the ring complex always faces towards the 5’-end of the newly synthesized DNA, while the other faces towards its 3’-end. Kawasoe et al. propose that this inbuilt asymmetry is used to direct the enzymes that correct mismatches towards the new strand and not the template strand.

Kawasoe et al. also found that MutSα strongly encourages the PCNA clamp to remain loaded on the DNA. They showed that this effect was much weaker if MutSα lacked the domain that it uses to interact with PCNA (Figure 1C). These findings further suggest that the interaction between MutSα and PCNA might act in favor of MMR, rather than DNA replication. If, as Kawasoe et al. suggest, the asymmetric PCNA-DNA complex forms a biological “memory” of which DNA strand is which, then the interaction between MutSα and PCNA appears to make that memory more stable over time, similar to converting short-term memories to long-term ones.

However, several questions remain to be answered. For example, the PCNA asymmetry and its interaction with MutSα may create a long-term “memory” for strand-specific MMR, but why do cells delay the packing of DNA into nucleosomes? Also, do the various modifications that are made to PCNA (such as ubiquitination and phosphorylation; Ortega et al., 2015) alter its role in strand-discrimination in MMR? Finally, does MMR in bacteria also use β clamp (the bacterial counterpart of PCNA) in the same way (Simmons et al., 2008)? Additional studies are now needed to answer these important questions.
